# Population Genetic Structure and Phylogeography of *Camellia flavida* (Theaceae) Based on Chloroplast and Nuclear DNA Sequences

**DOI:** 10.3389/fpls.2017.00718

**Published:** 2017-05-19

**Authors:** Su-Juan Wei, Yong-Bin Lu, Quan-Qing Ye, Shao-Qing Tang

**Affiliations:** ^1^Key Laboratory of Ecology of Rare and Endangered Species and Environmental Protection, Ministry of Education, Guangxi Normal UniversityGuilin, China; ^2^College of Life Science, Guangxi Normal UniversityGuilin, China

**Keywords:** *Camellia flavida*, phylogeography, species delimitation, genetic differentiation, habitat heterogeneity, conservation implication

## Abstract

*Camellia flavida* is an endangered species of yellow camellia growing in limestone mountains in southwest China. The current classification of *C. flavida* into two varieties, var. *flavida* and var. *patens*, is controversial. We conducted a genetic analysis of *C. flavida* to determine its taxonomic structure. A total of 188 individual plants from 20 populations across the entire distribution range in southwest China were analyzed using two DNA fragments: a chloroplast DNA fragment from the small single copy region and a single-copy nuclear gene called phenylalanine ammonia-lyase (PAL). Sequences from both chloroplast and nuclear DNA were highly diverse; with high levels of genetic differentiation and restricted gene flow. This result can be attributed to the high habitat heterogeneity in limestone karst, which isolates *C. flavida* populations from each other. Our nuclear DNA results demonstrate that there are three differentiated groups within *C. flavida*: var. *flavida* 1, var. *flavida* 2, and var. *patens*. These genetic groupings are consistent with the morphological characteristics of the plants. We suggest that the samples included in this study constitute three taxa and the var. *flavida* 2 group is the genuine *C. flavida*. The three groups should be recognized as three management units for conservation concerns.

## Introduction

*Camellia* (Theaceae) species with yellow flowers, known as yellow camellia, grow in parts of south China and north Vietnam. Studies have reported 10–16 species in China (Chang and Ren, 1998; Ming and Bartholomew, 2007). These plants are mainly distributed in Guangxi, with only a few reaching Guizhou and Yunnan Province (Chang and Ren, 1998; Liang, 2007). Most species of yellow camellia in China have restricted distributions. Yellow camellia frequently grows in primary forests, as understory shrubs or small trees, but they can also be found among secondary forests and shrubs that have suffered from deforestation. Some species grow in calcareous soil, and others are found in acidic soil. No known species naturally grows in both calcareous and acidic soil. Calcareous species are usually found at the bottom of depressions or on slopes in areas with high humidity and shade (Su and Mo, 1988; Su, 1994).

Yellow camellia are valuable ornamental plants and genetic resources for breeding. Moreover, yellow camellia leaves and flowers are used in traditional Chinese medicine and commercially available teas (He et al., 2016). Thousands of hectares are dedicated to growing yellow camellia for a variety of products. Excessive collecting from natural populations for ornamental planting has caused the destruction of wild populations, and all yellow camellia species were recently categorized as critically endangered, endangered, or near endangered species in the China Biodiversity Red List (data available at http://english.mep.gov.cn).

The karst area in southwestern China is the largest karst ecosystem in the world (Yuan, 1991; Wang et al., 2004). The karst is home to many plant species and contributes significantly to the floristic diversity of China, but many species are threatened (Orme et al., 2005; Hao et al., 2014; Luo et al., 2016). *Camellia flavida* Chang, one of yellow camellia, is a typical limestone species distributed in this area. It was first reported by Chang (1981), who found it in Longzhou. Since then, several new yellow camellia species collected from limestone mountains in the area have been described, including *Camellia longgangensis* (Liang and Mo, 1982), *Camellia longgangensis* var. *grandis* (Liang and Mo, 1982), *Camellia ptilosperma* (Liang, 1984), and *Camellia longruiensis* (Liang, 1993). These species are morphologically similar and were revised and treated as synonyms of *C. flavida* by Ming and Zhang (1993), Ming (2000), and Ming and Bartholomew (2007); however, Chang and Ren (1998) treated *C. longgangensis* as a synonym of *C. flavida* and classified the other three taxa as a distinct species, *C. grandis* (Table 1). Based on the herbarium specimens collected from limestone hills in Fusui and Wuming, adjacent to Longzhou, another four yellow camellia taxa were described, including *Camellia longgangensis* var. *patens* (Mo and Zhong, 1985), *Camellia quinqueloculosa* (Mo and Zhong, 1985), *Camellia multipetala* (Liang, 1993), and *Camellia wumingensis* (Liang, 1993). These were all treated as varieties of *C. flavida* (i.e., var. *patens*) according to the classification by Ming and Bartholomew (2007); however, Chang and Ren (1998) treated *C. quinqueloculosa* as a synonym of *Camellia aurea* and did not address the other three taxa (Table 1). Hence, there is disagreement regarding the classification of *C. flavida* among *Camellia* researchers.

**Table 1:** **Previous researchers' classifications of ***C. flavida*****.

**First attributed species name**	**Chang, 1991; Chang and Ren, 1998**	**Ming and Bartholomew, 2007**	**Ye and Xue, 2013**
*C. flavida*	*C. flavida*	*C. flavida*	–
*C. longgangensis*	*C. flavida*	*C. flavida* var. *flavida*	*C. longgangensis*
*C. longgangensis* var. *grandis*	*C. grandis*	*C. flavida* var. *flavida*	–
*C. ptilosperma*	*C. grandis*	*C. flavida* var. *flavida*	*C. longgangensis*
*C. longruiensis*	–	*C. flavida* var. *flavida*	*C. longgangensis*
*C. longgangensis* var. *patens*	–	*C. flavida* var. *patens*	*C. quinqueloculosa*
*C. quinqueloculosa*	*C. aurea*	*C. flavida* var. *patens*	*C. quinqueloculosa*
*C. multipetala*	–	*C. flavida* var. *patens*	*C. quinqueloculosa*
*C. wumingensis*	–	C. *flavida* var. *patens*	*C. wumingensis*

–, *athe species has not been treated by the researcher*.

Ye and Xue (2013) studied the morphological characteristics of the flowers, fruits, seeds, and leaves of the taxa that had been reduced to *C. flavida*, and the results suggested that (1) *C. ptilosperma* and *C. longruiensis* should be considered synonyms of *C. longgangensis*, (2) *C. longgangensis* var. *patens* and *C. multipetala* should be classified as a synonym of *C. quinqueloculosa*, and (3) *C. wumingensis* is an independent species (Table 1).

The size of the natural population of *C. flavida*, especially its variant *C. flavida* var. *patens*, has declined dramatically due to illegal transplanting. Thus, its distribution has become significantly fragmented. *C. flavida* has been listed as an endangered species in the China Species Red List (Wang and Xie, 2004) and China Biodiversity Red List (data available at http://english.mep.gov.cn). Effective conservation of endangered species requires accurate taxonomic classification, and incorrect taxonomy can lead to erroneous conservation decisions (Hong, 2016; Su et al., 2017). Genetic diversity is also critical to both the long-term survival of populations or species and their evolutionary potential (Frankel, 1974; Pérez-Espona and Consortium, 2017). Analysis of the spatial distribution of the genetic diversity of a species can help identify distinct genetic groups (Allendorf and Luikart, 2007; Mkare et al., 2017). However, the classification of *C. flavida* is still controversial, and the genetic diversity and population structure are not yet understood.

Molecular genetics analyses can help resolve the taxonomic uncertainties and define management units within species (Frankham et al., 2002), and therefore, develop an efficient strategy for conservation. In the present study, we conducted phylogeographic and population genetic analyses of *C. flavida* using both chloroplast DNA (cpDNA) and a single-copy nuclear gene. Plastid and nuclear markers are frequently used in phylogeographic and population genetic studies, as they present different features (Avise, 2009; Leuzinger et al., 2015). By combining these two types of markers, we aimed to (1) determine the population genetic structure and phylogeographic patterns of *C. flavida*, (2) clarify the species classification and boundaries, and (3) propose recommendations for guiding future preservation actions.

## Materials and methods

### Sample collection strategy and DNA isolation

**Figure 1:**
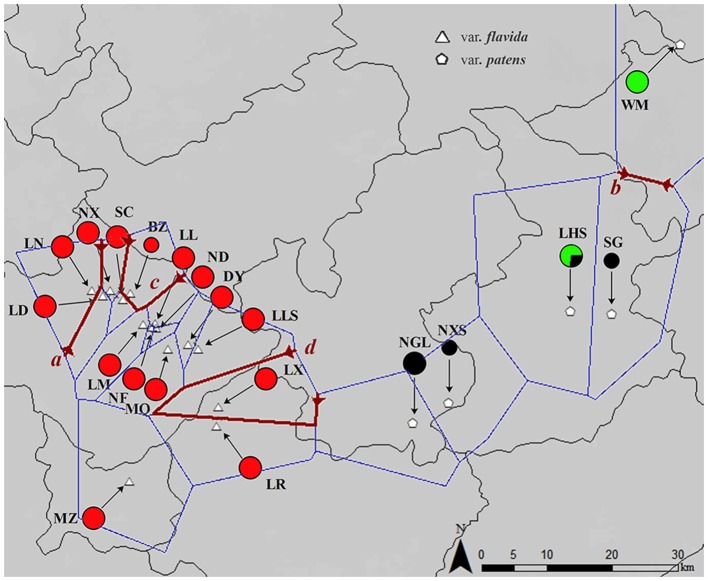
**Map of the sampling location and results of the barrier analyses**. The colors of the populations represent the cpDNA haplotype lineages as identified by phylogenetic analyses. The first four barriers *a, b, c*, and *d* defined by the Monmonier algorithm of the *PAL* datasets are represented by dark red lines.

Samples were collected for this study using the taxonomic classification of Ming and Bartholomew (2007), which was based on leaves and flowers. The 188 samples included in the analysis were collected from 20 wild populations from almost the entire geographical range of the species in southwest Guangxi, China (Figure 1). Ten individuals were selected per population. For those populations (BZ, SG, and NXS) containing fewer than 10 individuals, samples were collected from all available plants (Table 2). For each population, fresh leaves were randomly collected and dried in silica gel. Genomic DNA was extracted from dried leaves using a modified cetyl trimethylammonium bromide (CTAB) method (Doyle and Doyle, 1987). This modification to the CTAB protocol included incubation for 2 h in a 65°C water bath.

**Table 2:** **Geographical location, sample size, and genetic diversity of ***C. flavida*** based on cpDNA and ***PAL*** sequences**.

**Population code**	**Latitude (N)/Longitude (E)**	**Altitude (m)**	**Sample size cpDNA (*PAL*)**	**Number of haplotypes cpDNA (*PAL*)**	**Haplotype diversity cpDNA (*PAL*)**	**Nucleotide diversity cpDNA (*PAL*)**
MZ	106°52′/22°07′	343	10 (10)	1 (5)	0 (0.69474)	0 (0.00642)
LLS	107°02′/22°25′	202	10 (10)	1 (10)	0 (0.92105)	0 (0.00783)
DY	107°00′/22°25′	291	10 (10)	1 (7)	0 (0.83684)	0 (0.00811)
LL	106°56′22°28′	191	10 (10)	1 (9)	0 (0.90000)	0 (0.00580)
ND	106°56′/22°27′	247	10 (10)	1 (8)	0 (0.84737)	0 (0.00640)
LR	107°04′/22°14′	215	10 (10)	1 (6)	0 (0.79474)	0 (0.00418)
LM	106°54′/22°28′	213	10 (10)	1 (6)	0 (0.77895)	0 (0.00685)
LD	106°49′/22°32′	234	10 (10)	1 (9)	0 (0.87895)	0 (0.00760)
MQ	106°58′/22°25′	192	10 (10)	1 (5)	0 (0.73158)	0 (0.00732)
NF	106°55′/22°27′	176	10 (10)	1 (5)	0 (0.73158)	0 (0.00587)
NX	106°50′/22°32′	252	10 (10)	1 (5)	0 (0.66842)	0 (0.00640)
SC	106°51′/22°31′	357	10 (10)	1 (10)	0 (0.92105)	0 (0.00968)
var. *flavida* 1				11 (58)	0.91036 (0.97242)	0.00090 (0.00874)
BZ	106°52′/22°32′	257	6 (6)	1 (3)	0 (0.59091)	0 (0.00107)
LX	107°04′/22°17′	152	10 (9)	1 (6)	0 (0.66667)	0 (0.00269)
LN	106°47′/22°32′	368	10 (10)	1 (3)	0 (0.57368)	0 (0.00096)
var. *flavida* 2				3 (9)	0.67692 (0.82449)	0.00029 (0.00214)
var. *flavida*			146 (145)	14 (67)	0.92962 (0.97599)	0.00092 (0.00902)
SG	107°56′/22°29′	165	6 (5)	1 (5)	0 (0.66667)	0 (0.00347)
LHS	107°51′/22°30′	162	10 (10)	2 (5)	0.46667 (0.82105)	0.0009 (0.00571)
NXS	107°35′/22°18′	246	6 (6)	1 (5)	0 (0.75758)	0 (0.00306)
NGL	107°30′/22°15′	202	10 (10)	1 (8)	0 (0.74737)	0 (0.00390)
WM	108°06′/23°05′	138	10 (10)	1 (3)	0 (0.27895)	0 (0.00044)
var. *patens*			42 (41)	3 (21)	0.66086 (0.89852)	0.00090 (0.00455)
Total			188 (186)	17 (87)	0.94101 (0.97993)	0.00157 (0.00880)

### PCR amplification and DNA sequencing

A chloroplast genome fragment from the small single-copy (SSC) region containing several genes was amplified using the following three primer pairs: SSC1, CP30 (Xi et al., 2012), and SSC3. PCR products were sequenced in both directions with the same primers and four internal sequencing primers (SSC1-1, CP30-1, CP30-2, and SSC3-1). The single-copy nuclear gene phenylalanine ammonia-lyase (*PAL*) was amplified and sequenced using a primer design based on the sequences from *Camellia taliensis* (Liu et al., 2012). The sequences of primers used in this study are given in Table 3.

**Table 3:** **Primers used to amplify and sequence DNA from ***C. flavida*****.

**Primer**	**Sequence (5′–3′)**	**Source**	***T*a (°C)**
**CHLOROPLAST MARKERS**			
SSC1-F	AAACAGAAGAGATCCGAGT	Designed by the authors	51
SSC1-1	AATAGGCTATACTGACTGAA		
SSC1-R	TGTTATTTTGTGTGCCGTTC		
CP30-F	GTAGCAGCATGTATAAGAGCTGAA	Xi et al., 2012	57.5
CP30-1	AGCCCACATACGACGAAGTT		
CP30-2	TTTATTAGTAGGTCGATGAG		
CP30-R	GCTACTCGGACTCGAACCGAGAT		
SSC3-F	AAATCTCTTTCAACCGGAA	Designed by the authors	49
SSC3-1	GATAGAACTATCCAGTTACA		
SSC3-R	GCTCTATTTTGTTTATATTCGTC		
**NUCLEAR MARKERS**			
*PAL*-F	CACGTTACCACATTCAGCAACA	Designed by the authors	56
*PAL*-R	CCGCGAAACATCGATTAAGGG		

Both chloroplast and *PAL* DNA fragments were amplified in 50 μL reaction mixtures containing 30–50 ng genomic DNA, 5.0 μL of 10× PCR buffer (Mg^2+^ plus), 5 μL of dNTP mix (10 mM), 0.5 μL of each primer (50 μM), and 2.5 U of LA Taq DNA polymerase (all reagents, other than template DNA, from TaKaRa, China). DNA was amplified in a thermal cycler (Applied Biosystems, USA), programmed for an initial denaturation at 94°C for 3 min; followed by 30 cycles of 30 s denaturation at 94°C, 30 s annealing at various annealing temperatures (*T*a; Table 3), 1 min elongation at 72°C; and an additional extension for 10 min at 72°C. PCR products were purified and sequenced by Thermo Fisher Scientific (Guangzhou, China).

Previously reported karyotype analysis demonstrated that *C. flavida* is diploid (2*n* = 30) (Zhang and Ming, 1995). *PAL* sequence chromatograms containing double peaks at polymorphic sites were regarded as heterozygotes. For sequences with a single heterozygous site, we determined haplotypes using the method described by Clark (1990). For sequences with two or more additional peaks, the sequences of the two *PAL* haplotypes were analyzed by cloning and sequencing of PCR amplicons. *PAL* fragments were amplified with the high-fidelity DNA polymerase (PrimeSTAR HS DNA Polymerase, TaKaRa, China). Cloned PCR products were purified and sequenced by Sangon Biotech (Shanghai, China). At least four clones were sequenced per PCR product (maximum eight clones). All haplotype sequences in this study were deposited in GenBank: KX751947–KX751960, KU669063, KU669073, KU669071, and KX751961–KX752048.

### Data analyses

DNA sequences were aligned using CLUSTAL X (Thompson et al., 1997) and edited manually in BioEdit7.0.1 where necessary (Hall, 1999). Insertions or deletions (indels) in cpDNA were treated as substitutions (single events) (Caicedo and Schaal, 2004). The degree of nucleotide substitution saturation for the *PAL* gene was tested using DAMBE software (Xia and Lemey, 2009).

The global and population numbers of haplotypes (A), the gene diversity (h) (Nei, 1987), and the nucleotide diversity (π) (Tajima, 1983) were calculated using DNAsp v5 (Librado and Rozas, 2009). The median-joining method (Bandelt et al., 1999) was used to construct networks with Network 5.0 (Fluxus Technology Ltd. at www.Fluxus-engineering.com). A post-processing MP calculation was used to search for the shortest tree. Several values of the parameter ε were explored, without significant changes in network topology. The topology presented was obtained at the default settings (ε = 5). The geographical distribution of populations was mapped using ArcMap GIS (ESRI, 2009).

Phylogenetic analyses of the identified haplotypes for each marker were performed. Maximum-likelihood (ML) tree generation and bootstrap analyses were performed with the program RAxML-HPC-SSE3 (Stamatakis, 2006). We found the best-scoring ML tree using a generalized time reversible plus gamma model of sequence evolution with 1,000 bootstrap replicates. Sequences of the cpDNA region of *Camellia oleifera* (JQ975031) and *Camellia pitardii* (KF156837) and nuclear sequences of *C. taliensis* H16 (JX161631) and H17 (JX161632) were used as outgroups for analysis of *C. flavida*.

The presence of phylogeographic structures was inferred by testing for significant differences between *G*_*ST*_ and *N*_*ST*_ using PermutCpSSR 1.2.1, with 1,000 permutations (Pons and Petit, 1996). Gene flow (*Nm*) calculated using DNAsp v5 was used to assess the degree of genetic differentiation between populations.

A spatial analysis of molecular variance (SAMOVA) was conducted with SAMOVA 2.0. This approach defines groups of populations that are geographically homogeneous and maximally differentiated from each other (Dupanloup et al., 2002). We tested values for K in the range of 2–19, and the initial condition was set to 100 with 10,000 iterations. The configuration with the largest associated *F*_*CT*_ value after the 100 independent simulated annealing processes is retained as the best grouping of populations. In addition, for nuclear DNA data, analysis of the Monmonier's algorithm was implemented with the BARRIER 2.2 program, which identifies possible barriers to gene flow among the most differentiated groups of populations, creating a Delaunay triangulation network to connect adjacent populations and, consequently, a Voronoi tessellation set (Manni et al., 2004).

Analyses of molecular variance (AMOVA) (Excoffier et al., 1992) were conducted using Arlequin 3.1 (Excoffier et al., 2005). Populations were grouped into two varieties. In addition, we repeated the AMOVA analyses with three genetic groups identified in this study. We performed AMOVAs to quantify the genetic variation at three hierarchical levels: (i) among populations, (ii) within populations, and (iii) among groups of populations identified by the three genetic clusters found in this study.

## Results

### Chloroplast DNA sequence variation

Alignment of cpDNA sequences from188 *C. flavida* produced a consensus sequence of 5,178 base pairs. Seventeen haplotypes were defined with 38 polymorphic sites, including 33 substitutions and five indels. Of the five detected indels, two were five base pairs, and the other three were six, seven, and 11 base pairs. Most haplotypes were observed in only one population (Table S1). Only one population (LHS) had two chorotypes (C_15, C_16), and no haplotypes were shared between the two varieties. Four haplotypes were shared by two populations: C_3 (LL, ND), C_15 (LHS, WM), C_16 (SG, LHS), and C_17 (NXS, NGL) (Table S1). Total haplotype and nucleotide diversities were 0.94101 and 0.00157, respectively (Table 2). We did not observe sequence variation within populations, other than within cpDNA in the LHS population (Table 2).

**Figure 2:**
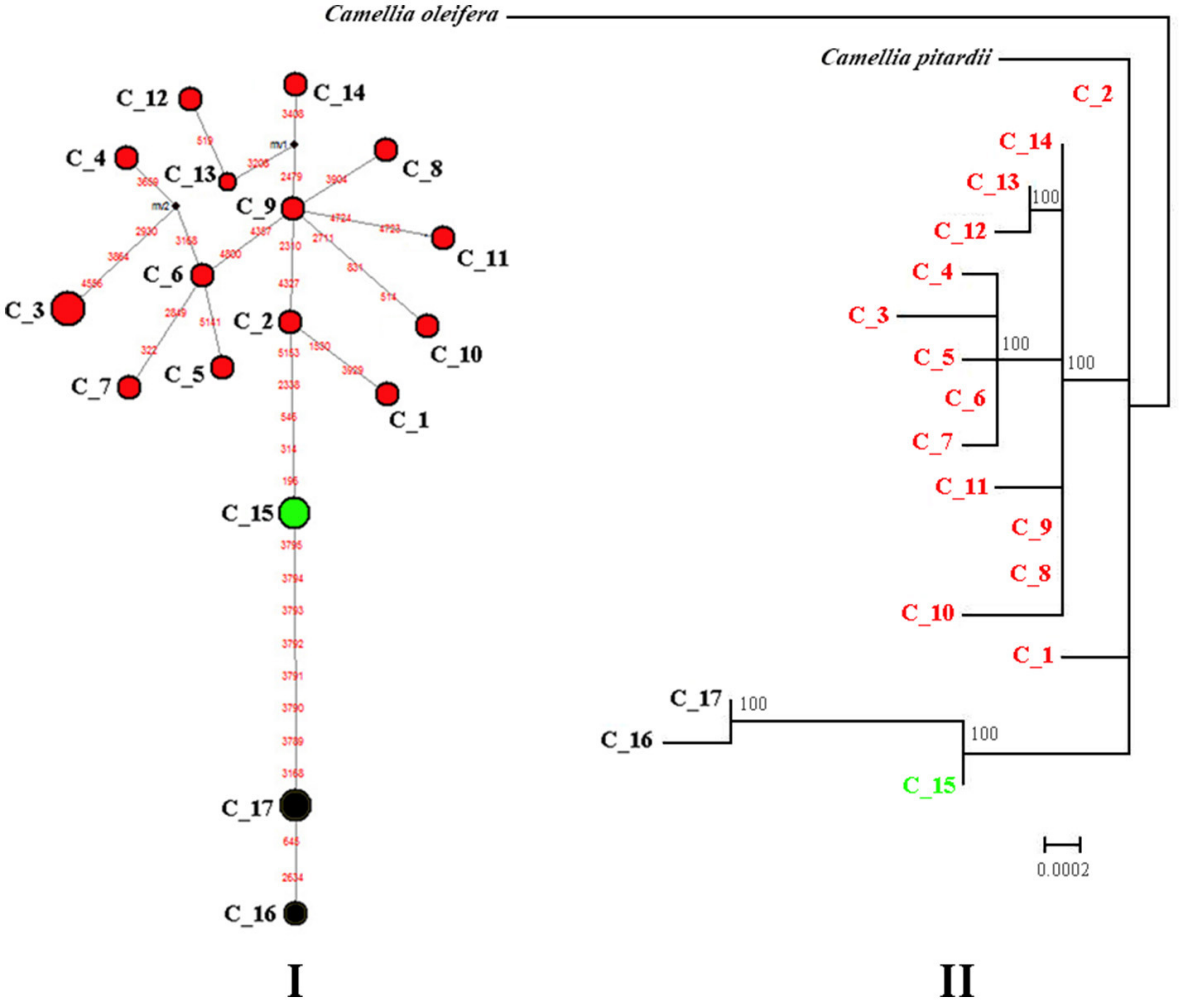
**Median-joining network (I) and Maximum Likelihood phylogenetic tree (II) showing the genetic relationships among the observed cpDNA haplotypes of ***C. flavida***. (I)** Median-joining network of 17 cpDNA haplotypes resolved in *C. flavida*. Each haplotype is designated by a number C_1 to C_17 (see Table S1). Colors denote the groups as identified by SAMOVA analyses of cpDNA marker. Circle size is proportional to haplotype frequency. Missing haplotypes are represented by black dots, and mutations are shown in red. **(II)** Maximum Likelihood tree. Numbers at nodes represent the result of the ML bootstrap analysis. Nodes without numbers correspond to supports weaker than 70% BP.

Median-joining network analysis was used to determine the relationships among cpDNA haplotypes and demonstrated that the two varieties are separated from one another by numerous mutations (Figure 2I). In var. *patens*, five populations were fixed for three haplotypes (C_15, C_16, and C_17). Haplotype C_15 was separated from C_16 and C_17 by a minimum of eight mutational steps (Figure 2I). The other part of the network contained all other haplotypes, clustered into a relatively concentrated group derived from var. *flavida* specimens. The ML tree produced a haplotype phylogenetic relationship similar to the one produced by the network analysis (Figures 2II).

The SAMOVA revealed that the cpDNA dataset of *C. flavida* can be partitioned into three groups. *F*_*CT*_ values increase slightly with K. When K was greater or equal to 4, at least one member of the group contained a single population of *C. flavida*, indicating that the group structure was disappearing. When K equaled 3, all var. *flavida* formed a group, and var. *patens* was separated into two groups (LHS and WM; SG, NXS and NGL) (Table S3).

The contribution of phylogenetic relationships between haplotypes to among-population differentiation was nonsignificant (*G*_*ST*_ = 0.976 and *N*_*ST*_ = 0.974, *N*_*ST*_ < *G*_*ST*_; *P* > 0.05). Low gene flow (*Nm* = 0.01) among the 20 *C. flavida* populations was detected by the cpDNA sequences.

### Nuclear DNA sequence variation

We calculated the average *I*_*SS*_ for subsets of 4, 8, 16, 32 OTUs (*I*_*SS*_ = 0.012, 0.011, 0.012, and 0.013, respectively) and found that they were significantly smaller than the corresponding *I*_*SS*.*C*_ values (*I*_*SS*.*C*_ = 0.805, 0.765, 0.744, and 0.718, respectively; *P* = 0.0000), indicating that substitution in the *PAL* gene region is not saturated. The alignment of 372 *PAL* gene sequences from 186 samples was 653 base pairs long and contained 52 parsimony informative sites, 17 single-nucleotide polymorphisms, and no indels, resulting in 87 distinct haplotypes (Table S2). Only one haplotype (H_67) was shared between var. *flavida* and var. *patens*. Each population contained both shared and unique haplotypes. Var. *flavida* had 67 haplotypes, 49 of which were unique. Var. *patens* had 21 haplotypes, of which 16 were unique and five were shared. WM was the only population with only unique haplotypes. Haplotype sharing usually occurred in adjacent populations, but some adjacent populations either did not share a haplotype at all (e.g., populations BZ and SC; populations LX and LR) or shared only very common haplotypes (e.g., populations DY and LLS). Total haplotype and nucleotide diversities were 0.97993 and 0.00880, respectively (Table 2). Haplotype diversity (*h*) ranged from 0.27895 to 0.92105, and nucleotide diversity (π) ranged from 0.00044 to 0.00968 (Table 2). The highest values of h and π were found in population SC (*h* = 0.92105, π = 0.00968), and the lowest were in population WM (*h* = 0.27895, π = 0.00044) (Table 2). Although levels of genetic diversity varied greatly, there is less obvious correlation between relationships measures of diversity in population level with geographical location.

In the SAMOVA analysis, *F*_*CT*_ values increased progressively as K was increased. For the first three *F*_*CT*_ values, the *F*_*CT*_ value was highest when K was 3. When K was between 4 and 19, each new group consisted of only a single population. Therefore, in our dataset, *C. flavida* populations can optimally be placed into three groups (Table S4). These three groups included group I (ND, MQ, NF, DY, LLS, LR, SC, NX, LM, LL, MZ, LD, named var. *flavida* 1), group II (BZ, LX, LN, named var. *flavida* 2), and group III (SG, LHS, NXS, NGL, WM, named var. *patens*).

**Figure 3:**
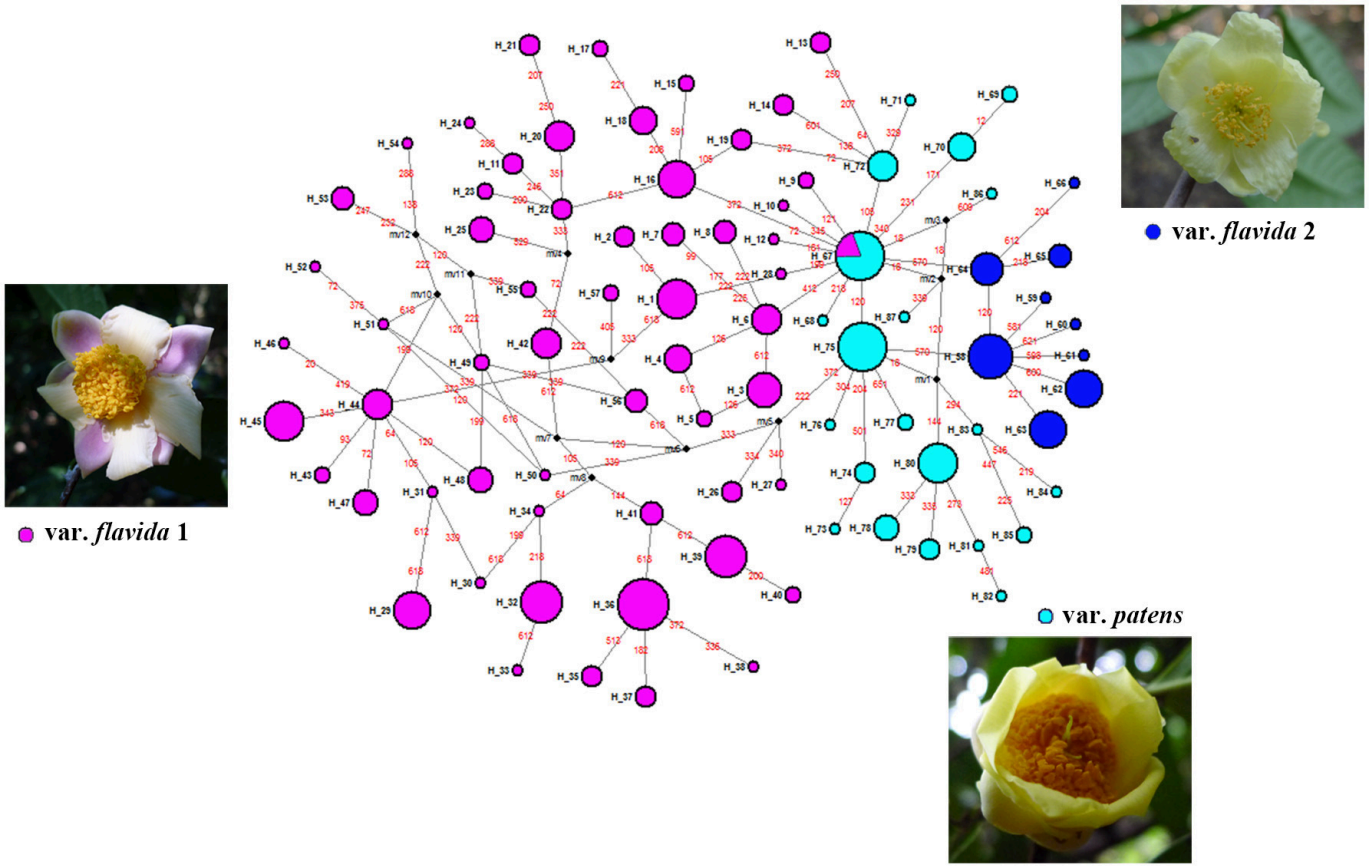
**Median-joining network for 87 ***PAL*** haplotypes and images of specimens of the three taxa identified in this study**. Each haplotype is designated by a number H_1 to H_89 (see Table S2). Colors denote the groups as identified by SAMOVA analyses of *PAL* marker. Circle size is proportional to haplotype frequency. Missing haplotypes are represented by black dots, and mutations are shown in red.

The *PAL* haplotype network is presented in Figure 3. Haplotypes from var. *patens* (H_67 through H_87) were clustered together and situated in the middle of the network, separating haplotypes of var. *flavida* 1 (H_1–H_57, H_67) from those of var. *flavida* 2 (H_58–H_66). In the ML tree, although the bootstrap value was low, all the haplotypes of var. *flavida* 2 and most haplotypes of var. *patens* clustered together respectively (Figure S1).

Our analyses of *PAL* sequence data revealed a phylogeographic structure across all populations (*G*_*ST*_ = 0.253, *N*_*ST*_ = 0.414; *N*_*ST*_ > *G*_*ST*_; *P* < 0.05). *Nm* value (0.35) detected by nuclear DNA sequences indicated that gene flow among populations was limited.

### Amova analysis

Over 60% of the genetic variation in cpDNA was attributable to variation among varieties; 57.95% of the variation was explained if the *C. flavida* populations were grouped into the three *PAL* SAMOVA groups (Table 4). The within population variation was low (1.76 and 2.18% in two varieties and the three *PAL* SAMOVA groups (Table 4). In contrast to the chloroplast DNA results, the molecular difference between var. *flavida* and var. *patens* in the *PAL* data was low (17.81%) and accounts for 53.28% of the genetic variation within populations (Table 4). When the populations were partitioned into the three *PAL* SAMOVA groups, a similar level of variation (51.58%) was observed within populations, but the variation among groups was much higher (31.19%). Thus, three genetic groups might be optimal for these populations.

**Table 4:** **Analyses of molecular variance (AMOVA) based on cpDNA and nuclear DNA from ***C. flavida*****.

		**Chloroplast DNA**	**Nuclear DNA**
**Grouping**	**Source of variation**	**Percentage of variation (%)**	**Percentage of variation (%)**
Varieties	Among varieties	63.46^**^	17.81^**^
	Among populations within varieties	34.78^**^	28.92^**^
	Within populations	1.76^**^	53.28^**^
SAMOVA groups	Among groups	57.95^**^	31.19^**^
	Among populations within groups	39.87^**^	17.23^**^
	Within populations	2.18^**^	51.58^**^
var. *flavida* 1	Among populations	100.00^**^	22.77^**^
	Within populations	0.00^**^	77.23^**^
var. *flavida* 2	Among populations	100.00^**^	32.90^**^
	Within populations	0.00^**^	67.10^**^
var. *patens*	Among populations	79.25^**^	31.68^**^
	Within populations	20.75^**^	68.32^**^

*A priori groupings were: (1) two varieties of C. flavida and (2) three SAMOVA groups of PAL marker, including var. flavida 1 (ND, MQ, NF, DY, LLS, LR, SC, NX, LM, LL, MZ, and LD); var. flavida 2 (BZ, LX, and LN); and var. patens (SG, LHS, NXS, NGL, and WM)*.

** *P < 0.001*.

The number of haplotypes (*A*), the haplotype diversity (*h*), and the nucleotide diversity (π) of each *PAL* SAMOVA group are shown in Table 2. The var. *flavida* 1 group had the highest level of genetic diversity (cpDNA: *A* = 11, *h* = 0.91036, π = 0.00090; *PAL*: *A* = 58, *h* = 0.97242, π = 0.00874). The var. *flavida* 2 group (cpDNA: *A* = 3, *h* = 0.67692, π = 0.00029; *PAL*: *A* = 9, *h* = 0.82449, π = 0.00214) and var. *patens* (cpDNA: *A* = 3, *h* = 0.66086, π = 0.00090; *PAL*: *A* = 21, *h* = 0.89852, π = 0.00455) have a relatively lower level of genetic diversity.

### Barrier to gene flow

In the barrier analysis of the *PAL* dataset (for graphical representation see Figures S2, S3 in Supplementary Material), we analyzed the first four barriers with (1) all populations and (2) the largest group (var. *flavida* 1). The Monmonier algorithm (barrier program) suggested four main barriers to gene flow in the distribution range of all populations (called *a* through *d*). Three barriers (*a, c*, and *d*) separate group var. *flavida* 2 populations (LN, BZ, and LX) from a group of var. *flavida* 1 populations (Figure 1). The second barrier (*b*) separates WM from SG (Figure 1). In the largest group (var. *flavida* 1), barriers were found in geographically close populations in addition to the barriers between distant populations (Figure S2).

## Discussion

### The samples collected constitute three taxa

According to nuclear DNA data, *C. flavida* is clustered into three genetic groups. These three groups are supported by the SAMOVA. Populations of var. *patens* formed a single genetic group. Haplotypes of the nuclear gene *PAL* from var. *patens* were phylogenetically related (Figure 3), with only one haplotype shared with var. *flavida*. Populations of var. *flavida* include two groups, var. *flavida* 1 and var. *flavida* 2. Although the distribution of var. *flavida* 1 and var. *flavida* 2 is overlapping, there are barriers to gene flow between these two groups (Figure 1). These three genetic groups are consistent with their morphological characteristics. The morphological characteristics of flowers, fruits, seeds, and leaves of nine populations of var. *flavida* 1 were studied by Ye and Xue (2013). The inner petals of this variant are light yellow, and the outer layer is light yellow with red patches or purple-red streaks (var. *flavida* 1, Figure 3). The plants flower from July to November. The LN and LX populations of var. *flavida* 2, are morphologically different from those of var. *flavida* 1. We found that the LN and LX petals were all light yellow, with no red or purple streaks or spots (var. *flavida* 2, Figure 3), and the plants mainly flowered from November to December. In addition, the shape of the leaves of var. *flavida* 2 is different from var. *flavida* 1, leading to the misidentification of var. *flavida* 2 as a different species by the administrator of Nonggang Nature Reserve, where these populations are located. According to the observations ofYe and Xue (2013), the petals of flowers from var. *patens* populations were all dark yellow, with no red or purple streaks or spots (Figure 3), and the plants flowered from January to February. In the SAMOVA analysis of chloroplast DNA sequences, the best value for K was three, but all var. *flavida* formed a group, and var. *patens* was separated into two groups. Similar results have been reported in many phylogeographic studies of closely related plant taxa. The distributions of chloroplast haplotypes frequently reveal geographic structure, and this geographic pattern may be incongruent with the current taxonomy (Rautenberg et al., 2010; Christe et al., 2014).

After combining molecular analyses and morphological observations, we concluded that the samples collected constitute three taxa, which is consistent with the three *PAL* groups. Samples from population BZ and the type herbarium specimens of *C. flavida* were collected from the same location; therefore, we suggest that var. *flavida* 2 is the genuine *C. flavida*.

### Genetic diversity and population structure

A high level of genetic diversity was observed at the species level in *C. flavida*. All measures of genetic diversity (haplotype numbers = 17, haplotype diversity = 0.94101, and nucleotide diversity = 0.00157 for cpDNA; haplotype numbers = 87, haplotype diversity = 0.97993, and nucleotide diversity = 0.00880 for the *PAL* gene) in *C. flavida* were higher than those in the congener, *C. taliensis*, which is believed to possess abundant variation (haplotype numbers = 12, haplotype diversity = 0.84129, and nucleotide diversity = 0.00314 for cpDNA; haplotype numbers = 17, haplotype diversity = 0.83639, and nucleotide diversity = 0.00417 for the *PAL* gene) (Liu et al., 2012). The number of *PAL* haplotypes observed in this study was five times higher than those identified in *C. taliensis*. The increased variation has several possible sources. First, the sampled populations include three genetic groups or taxa (Chang, 1991; Chang and Ren, 1998). Second, mutations and limited gene flow may have produced numerous unique haplotypes. At the variety level, the genetic diversity of var. *patens* was lower than that of var. *flavida*, either because var. *patens* has a smaller distribution range, a more isolated distribution, or smaller population sizes.

This study of 20 populations of *C. flavida* across its entire known geographic range revealed a very strong genetic structure. The population differentiation estimated from cpDNA was very high (*G*_*ST*_ = 0.976 and *N*_*ST*_ = 0.974), similar to plant species with the highest cpDNA differentiation (Petit et al., 2005). AMOVA analysis indicated that the among-groups variance is higher than that among populations within the two varieties. Chloroplast markers are haploid and are strictly maternally inherited in angiosperms (Birky, 2008). Therefore, they are expected to exhibit stronger genetic drift because their effective population size is lower than that of nuclear genes (Birky et al., 1983, 1989; Petit et al., 2005). In addition, substantial genetic differentiation of cpDNA may result from limited dispersal of heavy seeds that cannot travel long distances. We also discovered that many populations had low levels of genetic diversity in plastid DNA: Nineteen of the 20 analyzed populations were monomorphic (Table 2), suggesting an absence of gene flow through the movement of seeds (*Nm* = 0.01).

Population differentiation determined with the biparentally inherited nuclear *PAL* gene data was also high (*G*_*ST*_ = 0.253 and *N*_*ST*_ = 0.414) compared with that in other angiosperm species (mean *G*_*ST*_ = 0.183; Petit et al., 2005). The presence of significant phylogeographic structure was verified using the *PAL* data (*G*_*ST*_ < *N*_*ST*_, *P* < 0.05). AMOVA analysis indicated that most of the genetic diversity existed within populations (51.58%), but the among-groups variance (31.19%) is also very large. In our analysis of this nuclear marker, the gene flow (*Nm*) among populations was 0.35, and was primarily achieved by pollen transfer, given the lack of gene flow via seeds; however, gene flow between populations appears to be rather restricted. The mode of pollination and dispersal of *C. flavida* are poorly understood; however, bees are effective pollen vectors for its congeners *Camellia oleifera* (Deng et al., 2010) and *Camellia japonica* (Ueno et al., 2000). We also observed bees visiting *C. flavida* flowers during the field work for this study, and we suspect that bees may be important pollinators of *C. flavida*. The complex terrains of the karst provides a multitude of ecological niches and high habitat heterogeneity (Clements et al., 2006). Limestone karst landforms have been described as “terrestrial islands,” which are isolated, island-like areas on restricted land masses (Gao et al., 2015). *C. flavida* grows only on limestone hills in depressions containing thick soil layers, which exhibit typical characteristics of terrestrial islands; therefore, distribution of *C. flavida* is fragmented, and its populations may be isolated from one another. In addition, such distributions can also make it difficult for pollinators to locate flowers, which limit dispersal ranges and forms potential barriers to gene flow. The third barriers were found in geographically close populations in group I identified by the SAMOVA. This may explain the high levels of genetic differentiation detected in *C. flavida*.

### Implications for conservation

Incorrect species classification can combine several distinct species into one, which can jeopardize the protection of endangered species (Frankham et al., 2002). Our genetic data clearly show that var. *flavida* includes two distinct taxa. These two taxa should be regarded as two management units to be managed separately. Control of illegal harvesting is critical for conservation of these species, and *in situ* conservation measures should be established first. In var. *flavida* 2, there are only three populations. Thus, populations of BZ, LX, and LN should be candidates for *ex situ* conservation.

Within var. *patens*, no haplotype is shared among the three distribution points (Table S2) and high genetic divergence was detected. Because the number of populations is limited and there are few individuals in each population, these populations (NXS, NGL, LHS, SG, and WM) are reasonable candidates for *ex situ* conservation in germplasm banks.

## Conclusions

In our analysis of populations of a yellow Camellia species, we have found a high level of genetic differentiation and low gene flow attributable to the high habitat heterogeneity in limestone karst. There are three differentiated groups within the complex species. The detected genetic groups should be recognized as three conservation units.

## Author contributions

YL and QY collected population samples. SW performed experiments, analyzed the data, and wrote the manuscript. ST designed the study and wrote and revised the manuscript. All authors have read and approved the final manuscript.

## Funding

This study was supported by the National Natural Science Foundation of China (grant number 31260053) and Key Laboratory of Ecology of Rare and Endangered Species and Environmental Protection (Guangxi Normal University), Ministry of Education, China (grant number ERESEP2015Z01).

### Conflict of interest statement

The authors declare that the research was conducted in the absence of any commercial or financial relationships that could be construed as a potential conflict of interest.

## Supplementary material

Figure S1 **Maximum Likelihood phylogenetic tree reconstruction for the *C. flavida* based on *PAL* sequences**. Numbers at nodes represent the result of the ML bootstrap analysis. Nodes without numbers correspond to supports weaker than 70% BP. Double slashes on branches indicate branch length not in proportion.

Figure S2 **Delaunay triangulation and Voronoï tessellation of the barrier analyses for *C. flavida* across the entire study area**. Red points correspond to sampling sites. Barriers identified with the *PAL* dataset are dark red.

Figure S3 **Delaunay triangulation and Voronoï tessellation of the barrier analyses across a portion of the study area corresponding to populations containing individuals with the group (var. *flavida* 1) identified using *PAL* sequences**. Red points correspond to sampling sites; barriers identified with the *PAL* dataset are dark red.

Table S1 **Chloroplast haplotype distribution of 20 *C. flavida* populations**.

Table S2 ***PAL*** **haplotype distribution of 20** ***C. flavida*** **populations**.

Table S3 ***F***_***CT***_ **values for different numbers of population groups (K) inferred by the SAMOVA algorithm using the cpDNA dataset**.

Table S4 ***F***_***CT***_ **values for different numbers of population groups (K) inferred by the SAMOVA algorithm using the** ***PAL*** **dataset**.
