# Supplementary figures and images for: Population Genetic Structure and Phylogeography of Camellia flavida (Theaceae) Based on Chloroplast and Nuclear DNA Sequences
Source: Front Plant Sci. 2017 May 19;8:718. doi: 10.3389/fpls.2017.00718 (PMC5437371; doi:10.3389/fpls.2017.00718)

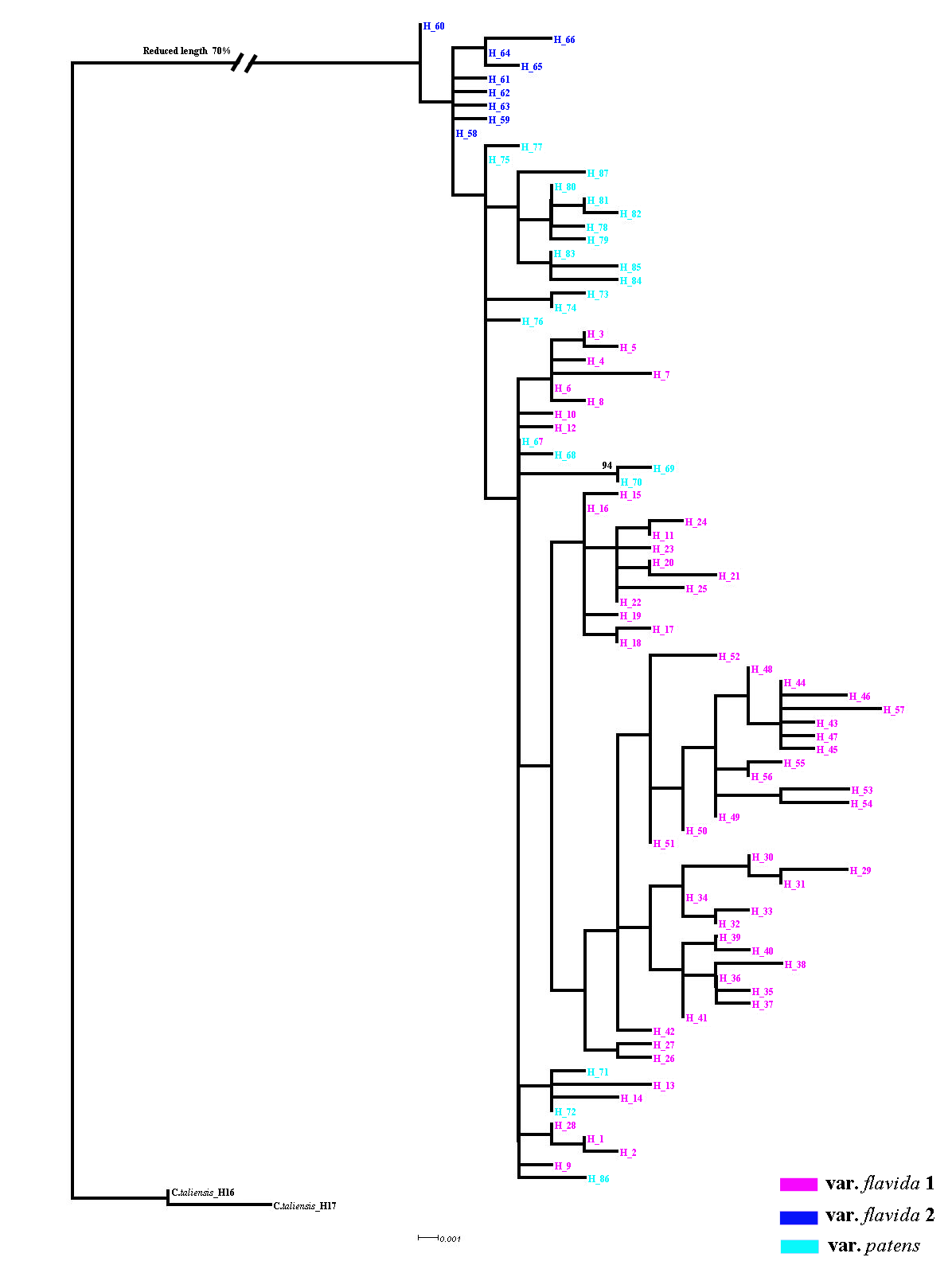

Supplement: Figure S1 — Maximum Likelihood phylogenetic tree reconstruction for the C. flavida based on PAL sequences. Numbers at nodes represent the result of the ML bootstrap analysis. Nodes without numbers correspond to supports weaker than 70% BP. Double slashes on branches indicate branch length not in proportion. [file SupplementaryFigureS1.TIF]
