# Supplementary material for: Population Genetic Structure and Phylogeography of Camellia flavida (Theaceae) Based on Chloroplast and Nuclear DNA Sequences
Source: Front Plant Sci. 2017 May 19;8:718. doi: 10.3389/fpls.2017.00718 (PMC5437371; doi:10.3389/fpls.2017.00718)
Supplement: Figure S2 — Delaunay triangulation and Voronoï tessellation of the barrier analyses for C. flavida across the entire study area. Red points correspond to sampling sites. Barriers identified with the PAL dataset are dark red. [file SupplementaryTablesandFigureS2-3.docx]

Table S1 Chloroplast haplotype distribution of 20 *C. flavida* populations.

| **Haplotypes** | **Populations** | | | | | | | | | | | | | | | | |  |  |  |
| --- | --- | --- | --- | --- | --- | --- | --- | --- | --- | --- | --- | --- | --- | --- | --- | --- | --- | --- | --- | --- |
|  | **MZ** | **LD** | **LL** | **ND** | **DY** | **NF** | **MQ** | **LLS** | **NX** | **SC** | **LR** | **LM** | **LN** | **BZ** | **LX** | **NXS** | **NGL** | **SG** | **LHS** | **WM** |
| **C_1** | **10** |  |  |  |  |  |  |  |  |  |  |  |  |  |  |  |  |  |  |  |
| **C_2** |  | **10** |  |  |  |  |  |  |  |  |  |  |  |  |  |  |  |  |  |  |
| **C_3** |  |  | **10** | **10** |  |  |  |  |  |  |  |  |  |  |  |  |  |  |  |  |
| **C_4** |  |  |  |  | **10** |  |  |  |  |  |  |  |  |  |  |  |  |  |  |  |
| **C_5** |  |  |  |  |  | **10** |  |  |  |  |  |  |  |  |  |  |  |  |  |  |
| **C_6** |  |  |  |  |  |  | **10** |  |  |  |  |  |  |  |  |  |  |  |  |  |
| **C_7** |  |  |  |  |  |  |  | **10** |  |  |  |  |  |  |  |  |  |  |  |  |
| **C_8** |  |  |  |  |  |  |  |  | **10** |  |  |  |  |  |  |  |  |  |  |  |
| **C_9** |  |  |  |  |  |  |  |  |  | **10** |  |  |  |  |  |  |  |  |  |  |
| **C_10** |  |  |  |  |  |  |  |  |  |  | **10** |  |  |  |  |  |  |  |  |  |
| **C_11** |  |  |  |  |  |  |  |  |  |  |  | **10** |  |  |  |  |  |  |  |  |
| **C_12** |  |  |  |  |  |  |  |  |  |  |  |  | **10** |  |  |  |  |  |  |  |
| **C_13** |  |  |  |  |  |  |  |  |  |  |  |  |  | **6** |  |  |  |  |  |  |
| **C_14** |  |  |  |  |  |  |  |  |  |  |  |  |  |  | **10** |  |  |  |  |  |
| **C_15** |  |  |  |  |  |  |  |  |  |  |  |  |  |  |  |  |  |  | **7** | **10** |
| **C_16** |  |  |  |  |  |  |  |  |  |  |  |  |  |  |  |  |  | **6** | **3** |  |
| **C_17** |  |  |  |  |  |  |  |  |  |  |  |  |  |  |  | **6** | **10** |  |  |  |

Table S2 *PAL* haplotype distribution of 20 *C. flavida* populations.

| **Haplotypes** | **Populations** | | | | | | | | | | | | | | | | |  |  |  |
| --- | --- | --- | --- | --- | --- | --- | --- | --- | --- | --- | --- | --- | --- | --- | --- | --- | --- | --- | --- | --- |
|  | **MZ** | **LD** | **LL** | **ND** | **DY** | **NF** | **MQ** | **LLS** | **NX** | **SC** | **LR** | **LM** | **LN** | **BZ** | **LX** | **NXS** | **NGL** | **SG** | **LHS** | **WM** |
| **H_1** |  | **5** |  |  |  | **2** |  |  |  | **2** |  | **2** |  |  |  |  |  |  |  |  |
| **H_2** |  |  | **2** |  |  |  |  |  |  |  |  | **1** |  |  |  |  |  |  |  |  |
| **H_3** |  |  |  |  |  | **1** | **8** |  |  |  |  |  |  |  |  |  |  |  |  |  |
| **H_4** |  |  |  | **6** |  |  |  |  |  |  |  |  |  |  |  |  |  |  |  |  |
| **H_5** |  |  |  | **2** |  |  |  |  |  |  |  |  |  |  |  |  |  |  |  |  |
| **H_6** |  |  |  | **5** | **1** |  |  | **1** |  |  |  |  |  |  |  |  |  |  |  |  |
| **H_7** |  |  |  |  |  |  |  |  |  | **4** |  |  |  |  |  |  |  |  |  |  |
| **H_8** |  |  |  |  |  |  |  |  | **4** |  |  |  |  |  |  |  |  |  |  |  |
| **H_9** |  |  |  |  |  |  |  |  |  |  | **2** |  |  |  |  |  |  |  |  |  |
| **H_10** |  |  |  |  |  |  |  | **1** |  |  |  |  |  |  |  |  |  |  |  |  |
| **H_11** |  |  |  |  |  |  |  | **3** |  |  |  |  |  |  |  |  |  |  |  |  |
| **H_12** |  |  |  |  |  |  |  |  |  | **1** |  |  |  |  |  |  |  |  |  |  |
| **H_13** |  |  |  |  | **3** |  |  |  |  |  |  |  |  |  |  |  |  |  |  |  |
| **H_14** | **3** |  |  |  |  |  |  |  |  |  |  |  |  |  |  |  |  |  |  |  |
| **H_15** |  | **2** |  |  |  |  |  |  |  |  |  |  |  |  |  |  |  |  |  |  |
| **H_16** |  |  |  |  |  |  | **2** |  |  | **1** | **7** |  |  |  |  |  |  |  |  |  |
| **H_17** |  |  |  |  |  |  |  |  |  |  | **2** |  |  |  |  |  |  |  |  |  |
| **H_18** |  |  |  |  |  |  |  |  |  |  | **6** |  |  |  |  |  |  |  |  |  |
| **H_19** |  |  |  |  | **3** |  |  |  |  |  |  |  |  |  |  |  |  |  |  |  |
| **H_20** |  |  |  |  | **7** |  |  |  |  |  |  |  |  |  |  |  |  |  |  |  |
| **H_21** |  |  |  |  | **3** |  |  |  |  |  |  |  |  |  |  |  |  |  |  |  |
| **H_22** |  | **1** |  |  |  |  |  |  | **2** |  |  |  |  |  |  |  |  |  |  |  |
| **H_23** |  |  |  |  |  |  |  |  |  | **2** |  |  |  |  |  |  |  |  |  |  |
| **H_24** | **1** |  |  |  |  |  |  |  |  |  |  |  |  |  |  |  |  |  |  |  |
| **H_25** |  |  |  |  |  |  |  |  |  |  |  | **5** |  |  |  |  |  |  |  |  |
| **H_26** |  |  |  |  |  |  |  | **3** |  |  |  |  |  |  |  |  |  |  |  |  |
| **H_27** |  |  | **1** |  |  |  |  |  |  |  |  |  |  |  |  |  |  |  |  |  |
| **H_28** |  | **1** |  |  |  |  |  |  |  |  |  |  |  |  |  |  |  |  |  |  |
| **H_29** |  |  | **2** |  |  |  | **7** |  |  | **1** |  |  |  |  |  |  |  |  |  |  |
| **H_30** |  |  |  | **1** |  |  |  |  |  |  |  |  |  |  |  |  |  |  |  |  |
| **H_31** | **1** |  |  |  |  |  |  |  |  |  |  |  |  |  |  |  |  |  |  |  |
| **H_32** |  |  | **3** |  |  | **7** |  |  |  |  |  | **3** |  |  |  |  |  |  |  |  |
| **H_33** |  |  | **1** |  |  |  |  |  |  |  |  |  |  |  |  |  |  |  |  |  |
| **H_34** |  |  |  | **1** |  |  |  |  |  |  |  |  |  |  |  |  |  |  |  |  |
| **H_35** |  |  |  |  |  |  |  |  |  | **3** |  |  |  |  |  |  |  |  |  |  |
| **H_36** |  |  |  |  |  |  |  |  | **11** |  |  | **8** |  |  |  |  |  |  |  |  |
| **H_37** |  |  |  |  |  |  |  |  |  | **3** |  |  |  |  |  |  |  |  |  |  |
| **H_38** |  |  |  |  |  |  | **1** |  |  |  |  |  |  |  |  |  |  |  |  |  |
| **H_39** |  | **1** | **1** |  |  | **8** |  |  |  | **2** |  | **1** |  |  |  |  |  |  |  |  |
| **H_40** |  | **2** |  |  |  |  |  |  |  |  |  |  |  |  |  |  |  |  |  |  |
| **H_41** |  |  | **3** | **1** |  |  |  |  |  |  |  |  |  |  |  |  |  |  |  |  |
| **H_42** |  |  | **5** |  |  |  | **2** |  |  |  |  |  |  |  |  |  |  |  |  |  |
| **H_43** |  |  |  |  | **2** |  |  |  |  |  |  |  |  |  |  |  |  |  |  |  |
| **H_44** |  | **2** |  | **3** |  | **2** |  |  |  |  |  |  |  |  |  |  |  |  |  |  |
| **H_45** | **10** |  |  |  |  |  |  | **2** |  |  |  |  |  |  |  |  |  |  |  |  |
| **H_46** |  |  |  |  | **1** |  |  |  |  |  |  |  |  |  |  |  |  |  |  |  |
| **H_47** | **5** |  |  |  |  |  |  |  |  |  |  |  |  |  |  |  |  |  |  |  |
| **H_48** |  | **5** |  |  |  |  |  |  |  |  |  |  |  |  |  |  |  |  |  |  |
| **H_49** |  |  |  |  |  |  |  |  |  |  | **2** |  |  |  |  |  |  |  |  |  |
| **H_50** |  |  |  |  |  |  |  |  |  |  | **1** |  |  |  |  |  |  |  |  |  |
| **H_51** |  |  |  | **1** |  |  |  |  |  |  |  |  |  |  |  |  |  |  |  |  |
| **H_52** |  |  |  |  |  |  |  |  | **1** |  |  |  |  |  |  |  |  |  |  |  |
| **H_53** |  | **1** |  |  |  |  |  | **1** | **2** |  |  |  |  |  |  |  |  |  |  |  |
| **H_54** |  |  |  |  |  |  |  | **1** |  |  |  |  |  |  |  |  |  |  |  |  |
| **H_55** |  |  |  |  |  |  |  | **2** |  |  |  |  |  |  |  |  |  |  |  |  |
| **H_56** |  |  |  |  |  |  |  | **4** |  |  |  |  |  |  |  |  |  |  |  |  |
| **H_57** |  |  |  |  |  |  |  | **2** |  |  |  |  |  |  |  |  |  |  |  |  |
| **H_58** |  |  |  |  |  |  |  |  |  |  |  |  | **9** | **4** | **1** |  |  |  |  |  |
| **H_59** |  |  |  |  |  |  |  |  |  |  |  |  |  |  | **1** |  |  |  |  |  |
| **H_60** |  |  |  |  |  |  |  |  |  |  |  |  |  |  | **1** |  |  |  |  |  |
| **H_61** |  |  |  |  |  |  |  |  |  |  |  |  |  | **1** |  |  |  |  |  |  |
| **H_62** |  |  |  |  |  |  |  |  |  |  |  |  |  |  | **10** |  |  |  |  |  |
| **H_63** |  |  |  |  |  |  |  |  |  |  |  |  | **10** |  |  |  |  |  |  |  |
| **H_64** |  |  |  |  |  |  |  |  |  |  |  |  | **1** | **7** |  |  |  |  |  |  |
| **H_65** |  |  |  |  |  |  |  |  |  |  |  |  |  |  | **4** |  |  |  |  |  |
| **H_66** |  |  |  |  |  |  |  |  |  |  |  |  |  |  | **1** |  |  |  |  |  |
| **H_67** |  |  | **2** |  |  |  |  |  |  | **1** |  |  |  |  |  | **4** | **10** |  |  |  |
| **H_68** |  |  |  |  |  |  |  |  |  |  |  |  |  |  |  | **1** |  |  |  |  |
| **H_69** |  |  |  |  |  |  |  |  |  |  |  |  |  |  |  |  |  |  | **2** |  |
| **H_70** |  |  |  |  |  |  |  |  |  |  |  |  |  |  |  |  |  | **1** | **5** |  |
| **H_71** |  |  |  |  |  |  |  |  |  |  |  |  |  |  |  |  | **1** |  |  |  |
| **H_72** |  |  |  |  |  |  |  |  |  |  |  |  |  |  |  | **5** | **2** |  |  |  |
| **H_73** |  |  |  |  |  |  |  |  |  |  |  |  |  |  |  |  | **1** |  |  |  |
| **H_74** |  |  |  |  |  |  |  |  |  |  |  |  |  |  |  | **1** | **2** |  |  |  |
| **H_75** |  |  |  |  |  |  |  |  |  |  |  |  |  |  |  |  |  |  |  | **17** |
| **H_76** |  |  |  |  |  |  |  |  |  |  |  |  |  |  |  |  |  |  |  | **1** |
| **H_77** |  |  |  |  |  |  |  |  |  |  |  |  |  |  |  |  |  |  |  | **2** |
| **H_78** |  |  |  |  |  |  |  |  |  |  |  |  |  |  |  |  |  |  | **5** |  |
| **H_79** |  |  |  |  |  |  |  |  |  |  |  |  |  |  |  |  |  |  | **3** |  |
| **H_80** |  |  |  |  |  |  |  |  |  |  |  |  |  |  |  |  |  | **6** | **5** |  |
| **H_81** |  |  |  |  |  |  |  |  |  |  |  |  |  |  |  |  |  | **1** |  |  |
| **H_82** |  |  |  |  |  |  |  |  |  |  |  |  |  |  |  |  |  | **1** |  |  |
| **H_83** |  |  |  |  |  |  |  |  |  |  |  |  |  |  |  |  | **1** |  |  |  |
| **H_84** |  |  |  |  |  |  |  |  |  |  |  |  |  |  |  | **1** |  |  |  |  |
| **H_85** |  |  |  |  |  |  |  |  |  |  |  |  |  |  |  |  | **2** |  |  |  |
| **H_86** |  |  |  |  |  |  |  |  |  |  |  |  |  |  |  |  | **1** |  |  |  |
| **H_87** |  |  |  |  |  |  |  |  |  |  |  |  |  |  |  |  |  | **1** |  |  |

Table S3 *F_CT_* values for different numbers of population groups (K) inferred by the SAMOVA algorithm using the cpDNA dataset.

| **K** | **Population grouping** | ***F_CT_*** | **P_value** |
| --- | --- | --- | --- |
| **k=2** | **(SG, NXS , NGL) (MZ, LD, LL, ND, DY, NF, MQ, LLS, NX, SC, LR, LM, LN, BZ, LX, LHS, WM )** | **0.67311** | **0.00000** |
| **k=3** | **(MZ, LD, LL, ND, DY, NF, MQ, LLS, NX, SC, LR, LM, LN, BZ, LX) (SG, NXS, NGL) (LHS, WM)** | **0.68230** | **0.00000** |
| **k=4** | **(SG, NXS, NGL) (MZ, LD, LL, DY, NF, MQ, LLS, NX, SC, LR, LM, LN, BZ, LX) (LHS, WM) (ND)** | **0.67438** | **0.00000** |
| **k=5** | **(SG, NXS, NGL) (ND, LL, DY, NF, MQ, LLS) (NX, SC, LR, LM, LN, BZ, LX) (LHS, WM) (MZ, LD)** | **0.68746** | **0.00000** |
| **k=6** | **(SG, NXS, NGL) (LL, DY, NF, MQ, LLS) (NX, SC, LR, LM, LN, BZ, LX) (LHS, WM) (MZ, LD) (ND)** | **0.71239** | **0.00000** |
| **k=7** | **(SG, NXS, NGL) (LL, DY, NF, MQ, LLS) (ND) (NX, SC, LM, LN, BZ, LX) (LHS, WM) (MZ, LD) (LR)** | **0.73483** | **0.00000** |
| **k=8** | **(SG, NXS, NGL) (DY, NF, MQ) (ND) (LL, LLS) (NX, SC, LM, LN, BZ, LX) (LHS, WM) (MZ, LD) (LR)** | **0.76048** | **0.00000** |
| **k=9** | **(SG, NXS, NGL) (DY, NF, MQ) (ND) (LL, LLS) (LD, NX, SC, LM) (LX, LN, BZ) (LHS, WM) (MZ) (LR)** | **0.77832** | **0.00000** |
| **K=10** | **(SG, NXS, NGL) (DY, NF, MQ) (ND) (LL, LLS) (LD, NX, SC, LM) (LX) (LN, BZ) (LHS, WM) (MZ) (LR)** | **0.79251** | **0.00000** |
| **K=11** | **(SG, NXS, NGL) (DY, NF, MQ) (ND) (LL, LLS) (LD, NX, SC) (LM) (LX) (LN, BZ) (LHS, WM) (MZ) (LR)** | **0.81585** | **0.00000** |
| **K=12** | **(SG) (NXS, NGL) (DY, NF, MQ) (ND) (LL, LLS) (LD, NX, SC) (LM) (LX) (LN, BZ) (LHS, WM) (MZ) (LR)** | **0.82736** | **0.00000** |
| **K=13** | **(SG) (NXS, NGL) (DY) (NF, MQ) (ND) (LL, LLS) (LD, NX, SC) (LM) (LX) (LN, BZ) (LHS, WM) (MZ) (LR)** | **0.85624** | **0.00000** |
| **K=14** | **(SG) (NXS, NGL) (DY) (NF, MQ) (ND) (LL, LLS) (LD, NX, SC) (BZ) (LM) (LX) (LN) (LHS, WM) (MZ) (LR)** | **0.85533** | **0.00000** |
| **K=15** | **(SG) (NXS) (NGL) (DY) (NF, MQ) (ND) (LL, LLS) (LD, SC) (NX) (LM) (LX) (LN BZ) (LHS, WM) (MZ) (LR)** | **0.85936** | **0.00000** |
| **K=16** | **(SG) (NXS, NGL) (DY) (NF, MQ) (ND) (LL, LLS) (LD) (NX) (LM) (LX) (LN) (SC) (BZ) (LHS, WM) (MZ) (LR)** | **0.91429** | **0.00000** |
| **K=17** | **(SG) (NXS, NGL) (DY) (NF, MQ) (ND) (LL, LLS) (LD) (NX) (LM) (LX) (LN) (SC) (BZ) (LHS) (WM) (MZ) (LR)** | **0.93079** | **0.00000** |
| **K=18** | **(SG) (NXS) (NGL) (DY) (NF, MQ ) (ND) (LL, LLS) (LD) (NX) (LM) (LX) (LN) (SC) (BZ) (LHS) (WM) (MZ) (LR)** | **0.91412** | **0.00000** |
| **K=19** | **(SG) (NXS, NGL) (DY) (NF) (MQ) (ND) (LL) (LLS) (LD) (NX) (LM) (LX) (LN) (SC) (BZ) (LHS) (WM) (MZ) (LR)** | **0.97414** | **0.00391** |

Table S4 *F_CT_* values for different numbers of population groups (K) inferred by the SAMOVA algorithm using the *PAL* dataset.

| **K** | **groups of populations** | ***F_CT_*** | **P-value** |
| --- | --- | --- | --- |
| **k=2** | **(ND, MQ, NF, DY, LLS, LR, SC, NX, LM, LL, MZ ,LD) (BZ, LX, LN, SG, LHS, NXS, NGL, WM)** | **0.29533** | **0.00000** |
| **k=3** | **(SG, LHS, NXS, NGL, WM) (ND, MQ, NF, DY, LLS, LR, SC, NX, LM, LL, MZ, LD) (BZ, LX, LN)** | **0.31190** | **0.00000** |
| **k=4** | **(BZ, LX, LN, WM) (ND, MQ, NF, DY, LLS, LR, SC, NX, LM, LL, MZ, LD) (NXS, NGL) (SG, LHS)** | **0.31120** | **0.00000** |
| **k=5** | **(BZ, LX, LN) (LR) (SG, LHS, NXS, NGL, WM) (ND, MQ, NF, DY, LLS, SC, NX, LM, LL, LD) (MZ)** | **0.31262** | **0.00000** |
| **k=6** | **(BZ, LX, LN, NXS, NGL, WM) (ND, MQ, NF, LLS, SC, NX, LM, LL, LD) (SG LHS) (DY) (MZ) (LR)** | **0.31322** | **0.00000** |
| **k=7** | **(MQ, NF, LLS, SC, NX, LM, LL, LD) (BZ, LX, LN) (SG, LHS, NXS, NGL, WM) (DY) (LR) (MZ) (ND)** | **0.31845** | **0.00000** |
| **k=8** | **(MQ, NF, SC, NX, LM, LL, LD) (SG, LHS, NXS, NGL, WM) (BZ, LX, LN) (LR) (ND) (MZ) (DY) (LLS)** | **0.31617** | **0.00000** |
| **k=9** | **(ND, MQ) (NF, LM, LL) (SG, LHS) (LLS, SC, NX, LD) (NXS, NGL, WM, LX) (BZ, LN) (MZ) (LR) (DY)** | **0.31855** | **0.00000** |
| **k=10** | **(MQ, NF, LLS, SC, NX, LM, LL, LD) (SG, LHS) (NXS, NGL) (BZ, LX) (LN) (WM) (ND) (MZ) (DY) (LR)** | **0.33171** | **0.00000** |
| **k=11** | **(NF, SC, LM, LL, NX, LD) (SG, LHS) (NXS, NGL, WM) (BZ LN) (LX) (LLS) (ND) (MQ) (MZ) (DY) (LR)** | **0.34113** | **0.00000** |
| **k=12** | **(LLS, LD) (NF, LM, LL, SC) (ND, MQ) (MZ) (DY) (SG, LHS) (NXS, NGL, WM) (BZ)) (LN) (LX) (NX) (LR)** | **0.34013** | **0.00000** |
| **k=13** | **(NF, LM, LL) (SC, LD, NX) (SG, LHS) (NXS, NGL) (BZ, LX) (LN) (LLS) (ND) (MQ) (MZ) (DY) (WM) (LR)** | **0.35868** | **0.00000** |
| **k=14** | **(NF, LL) (SC, NX, LM) (SG, LHS) (NXS, NGL, WM) (BZ) (LX) (LN) (LLS) (LD) (ND) (MQ) (MZ) (DY) (LR)** | **0.36295** | **0.00000** |
| **k=15** | **(NF, LL) (SC, NX, LM) (SG, LHS )(NXS, NGL) (WM) (BZ) (LX) (LN) (LLS) (LD) (ND) (MQ) (MZ) (DY) (LR)** | **0.37841** | **0.00000** |
| **k=16** | **( NF, LL, LM) (SG, LHS) (NXS, NGL) (LLS) (SC) (NX) (LD) (ND) (MQ) (MZ) (DY) (LR) (WM) (BZ) (LX) (LN)** | **0.38732** | **0.00000** |
| **k=17** | **(NF, LL) (NXS, NGL) (SG, LHS) (LM) (SC) (NX) (LD) (ND) (MQ) (MZ) (DY) (LR) (WM) (BZ) (LX) (LN) (LLS)** | **0.40118** | **0.00000** |
| **k=18** | **(NF, LL) (NXS, NGL) (LM) (SC) (NX) (LD) (ND) (MQ) (MZ) (DY) (SG) (LHS) (LR) (WM) (BZ) (LX) (LN) (LLS)** | **0.41221** | **0.00098** |
| **k=19** | **(NXS, NGL) (LLS) (NF) (LL) (LM) (SC) (NX) (LD) (ND) (MQ) (MZ) (DY) (SG) (LHS) (LR) (WM) (BZ) (LX) (LN)** | **0.41247** | **0.00293** |


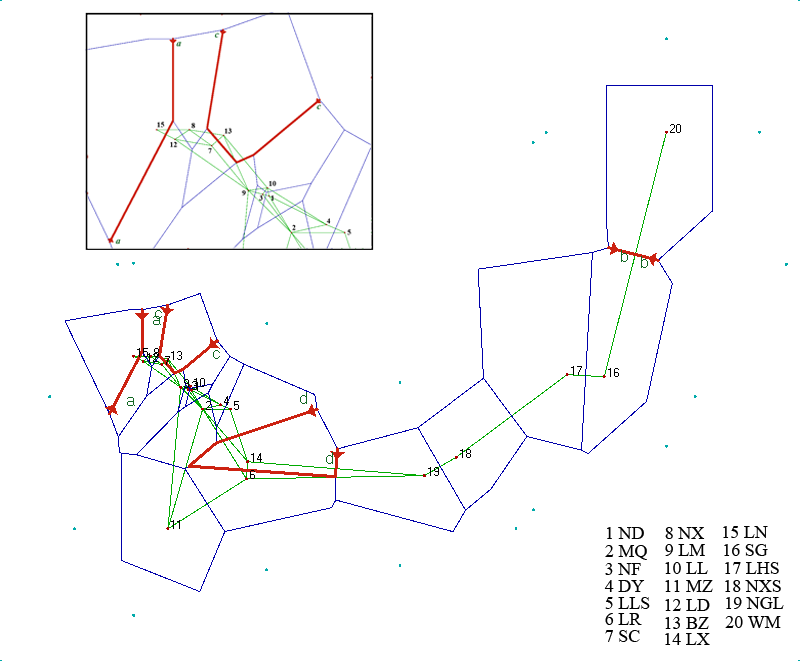


Figure S2: Delaunay triangulation and Voronoï tessellation of the barrier analyses for *C. flavida* across the entire study area*.* Red points correspond to sampling sites. Barriers identified with the *PAL* dataset are dark red.


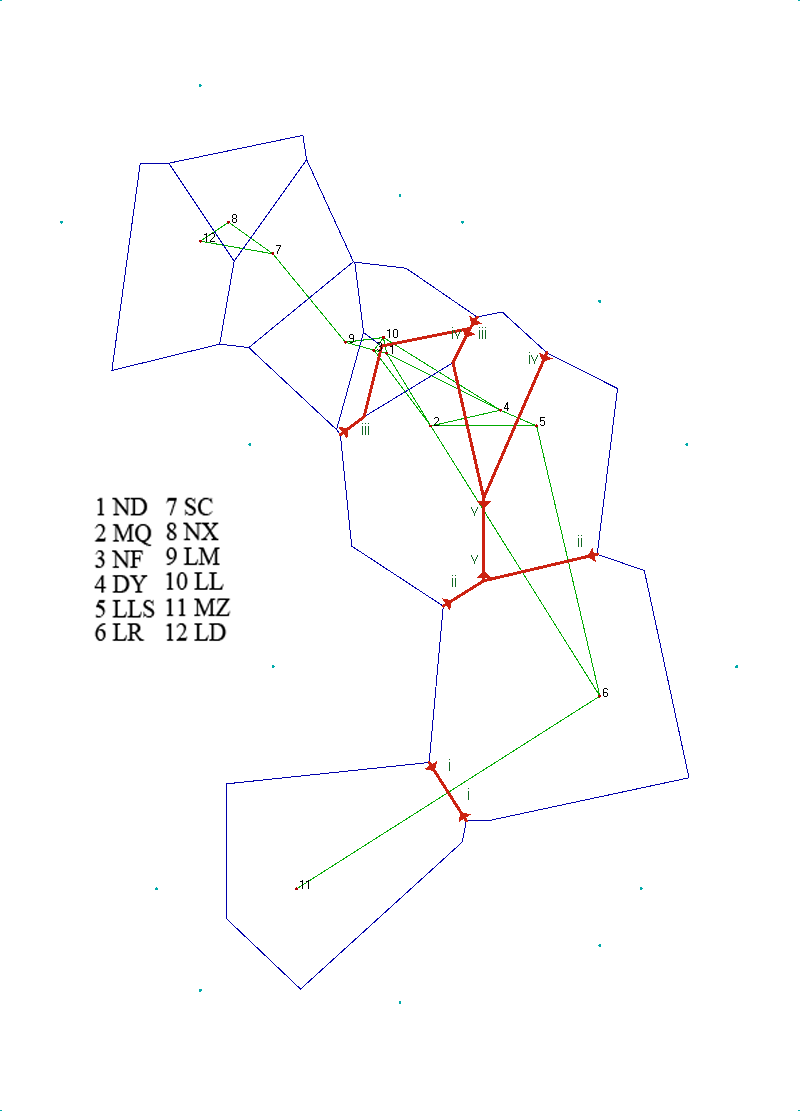


Figure S3: Delaunay triangulation and Voronoï tessellation of the barrier analyses across a portion of the study area corresponding to populations containing individuals with the group (var*.* *flavida* 1) identified using *PAL* sequences*.* Red points correspond to sampling sites; barriers identified with the *PAL* dataset are dark red.
